# Progress and Perspective for In Situ Studies of Oxygen Reduction Reaction in Proton Exchange Membrane Fuel Cells

**DOI:** 10.1002/advs.202300550

**Published:** 2023-04-25

**Authors:** Wenhui Zhao, Guangtong Xu, Wenyan Dong, Yiwei Zhang, Zipeng Zhao, Limei Qiu, Juncai Dong

**Affiliations:** ^1^ Sinopec Research Institute of Petroleum Processing Co., Ltd. Beijing 100083 P. R. China; ^2^ School of Materials Science and Engineering Beijing Institute of Technology Beijing 100081 P. R. China; ^3^ Beijing Synchrotron Radiation Facility Institute of High Energy Physics Chinese Academy of Sciences Beijing 100049 P. R. China

**Keywords:** electrocatalysis, in situ techniques, oxygen reduction reaction, proton exchange membrane fuel cell (PEMFC)

## Abstract

Proton exchange membrane fuel cell (PEMFC) is one of the most promising energy conversion devices with high efficiency and zero emission. However, oxygen reduction reaction (ORR) at the cathode is still the dominant limiting factor for the practical development of PEMFC due to its sluggish kinetics and the vulnerability of ORR catalysts under harsh operating conditions. Thus, the development of high‐performance ORR catalysts is essential and requires a better understanding of the underlying ORR mechanism and the failure mechanisms of ORR catalysts with in situ characterization techniques. This review starts with the introduction of in situ techniques that have been used in the research of the ORR processes, including the principle of the techniques, the design of the in situ cells, and the application of the techniques. Then the in situ studies of the ORR mechanism as well as the failure mechanisms of ORR catalysts in terms of Pt nanoparticle degradation, Pt oxidation, and poisoning by air contaminants are elaborated. Furthermore, the development of high‐performance ORR catalysts with high activity, anti‐oxidation ability, and toxic‐resistance guided by the aforementioned mechanisms and other in situ studies are outlined. Finally, the prospects and challenges for in situ studies of ORR in the future are proposed.

## Graphical abstract

Herein, the in situ studies of oxygen reduction reaction (ORR) in the cathode of proton exchange membrane fuel cells are summarized, including the introduction of relevant in situ techniques, the reaction and catalyst failure mechanism, and the development of advanced catalysts guided by in situ studies. The challenges and perspectives are proposed for the in situ study of ORR.

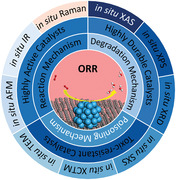

## 1 Introduction

The gradual exhaustion of fossil energy and increasing environmental pollution have become critical issues in today's world. Hydrogen is regarded as one of the most promising alternative clean and renewable energy in the future. One of the most effective ways to utilize hydrogen is proton exchange membrane fuel cells (PEMFCs) which can directly convert chemical energy stored in hydrogen into electrical energy with high conversion efficiency, zero‐emission, and moderate operating temperatures.^[^ ^1^, ^2^, ^3^ ^]^

However, there are still some critical issues that need to be addressed to expand the commercialization of PEMFCs. One of the most significant obstacles is the high cost of the only practical Pt‐based catalysts, which make up to more than 40% of the total fuel cell stack cost.^[^ ^4^ ^]^ Particularly, the kinetic sluggish oxygen reduction reaction (ORR) in the cell cathode raises the consumption of Pt which has made the main contribution to the high cost of catalysts. To cut the cost, it is indispensable to develop highly active ORR catalysts with low Pt loading and the U.S. Department of Energy (DOE) has announced a mass activity target of 0.44 A/mg_Pt_ @ 0.9 V_iR‐free_.^[^ ^5^ ^]^

On the other hand, to decrease the replacement cost of catalysts, a robust durability is also imperative, which is a challenge for ORR catalysts in the acidic and corrosive cathode environment. For instance, although commercial Pt/C catalyst is one of the most durable ORR catalysts, it would still experience Pt nanoparticle (NP) degradation processes due to the harsh conditions. Our group have revealed that the mean diameter of Pt NPs in a commercial Pt/C catalyst increased to four times of its original value after a 1800‐hour real vehicle running test.^[^ ^6^ ^]^ In addition, some specific operations of PEMFC might bring about extreme conditions in the cathode and consequently trigger severer degradation of the catalysts. For example, the automotive operations of fuel cell vehicles, such as startup‐shutdown and open‐circuit/idling, can induce extraordinarily high potentials in the cathode, leading to the oxidation and dissolution of Pt catalyst.^[^ ^7^ ^]^ The DOE has also defined the requirement for the durability under different operation modes that can be simulated by prescriptive accelerated degradation tests (ADTs).^[^ ^5^ ^]^ Over the past decades, abundant efforts have been exerted to develop the cathode catalysts that could achieve the aforementioned DOE targets.^[^ ^8^, ^9^, ^10^, ^11^, ^12^ ^]^ However, there are few catalysts reaching the activity and durability targets simultaneously, and the performance of the catalysts has not been verified when applied in real PEMFC applications.

Moreover, since air is the generally used cathode‐side oxidant of the practical PEMFC applications, the airborne contaminants including SO_x_, NO_x_, and organic impurities, can poison the cathode catalysts and induce severe decay of PEMFC performance during operation.^[^ ^13^ ^]^ Consequently, it is also important to develop toxic‐resistant ORR catalysts to prolong their service life in practical applications and further reduce the replacement cost of the catalysts. However, the poisoning problem has not been sufficiently taken into account in the literature.

Obviously, developing ORR catalysts with high Pt mass activity, robust durability, and favorable toxic‐resistance is still an urgent call for the PEMFC industry. To provide valuable design rules for the high‐performance ORR catalysts, it is vital to study the reaction mechanism as well as the catalyst failure (e.g., the degradation and poisoning) mechanisms during operation. The overall reaction of oxygen reduction in the cathode of PEMFC is generally regarded as a four‐electron or two‐electron process, as depicted in Equations (1) and (2).

(1) $$O_{2}+4H^{+}+4e^{-}\to 2H_{2}O$$

(2) $$O_{2}+2H^{+}+2e^{-}\to H_{2}O_{2}$$

The complicated circumstances at the gas–solid–liquid interface in the cathode make the ORR a complex process involving several reactions and diverse pathways. Furthermore, the extreme conditions induced by automotive operations and the introduction of air contaminants make the process even more complex. The real‐time monitoring of the reaction will be of great help in understanding the complicated reaction and catalyst failure mechanisms.

Fortunately, plenty of in situ characterization techniques such as in situ X‐ray absorption spectroscopy (XAS), in situ X‐ray diffraction (XRD), in situ Fourier transformation infrared spectroscopy (FTIR), in situ Raman spectroscopy, in situ transmission electron microscopy (TEM), etc.^[^ ^14^, ^15^, ^16^, ^17^, ^18^ ^]^ have been developed to be applied in the research of electrocatalytic mechanisms and could provide abundant information including but not limited to oxidation state, local coordination, structural reconstruction, vibrational information, and morphological evolution in real‐time during the reaction. Those techniques implement the detection of intermediates as well as products and the monitoring of structural and electronic evolution of catalysts during the reaction processes, and thus facilitate the mechanism research.

Recently, some reviews have afforded comprehensive insights into the in situ study of electrocatalysis in terms of the in situ techniques that have been applied in electrocatalysis research, as well as the in situ study of a specific electrocatalytic reaction.^[^ ^18^, ^19^, ^20^ ^]^ However, there are few of them focusing on in situ study of ORR in PEMFCs which will be overviewed in the present review, from a practical perspective. We first introduce the in situ methods used in ORR research including the principle of the methods, the design of the in situ cells, and the application of the methods. Then the in situ study of ORR mechanism and the catalyst failure mechanisms, including Pt NP degradation, Pt oxidation, and poisoning by air contaminants are elaborated. The development of active, anti‐oxidation, and toxic‐resistant ORR catalysts with the guidance of the mechanisms above and some other in situ studies is also overviewed. It should be noted that the in situ study of the poisoning of ORR catalysts induced by air contaminants is highlighted which is of important practical significance but has got limited concern in the literature. Finally, the challenges and perspectives of future research are proposed, which could pave the way for further development of in situ study of ORR in PEMFCs.

Herein, the mechanism discussed is mainly about the ORR in acidic media or in a H_2_/O_2_ or H_2_/air PEMFC while the catalysts are mainly Pt‐based catalysts that have been or have the most potential to be commercially used.

## 2 Introduction to In Situ Characterization Techniques

This section introduces the in situ X‐ray based techniques that have been primarily used in ORR research, as well as the commonly used in situ microscopic techniques and in situ vibrational spectroscopies. The content covers the principles of these methods, the in situ cells used for them, and their application in ORR research.

### 2.1 X‐Ray‐Based In Situ Characterization Techniques

X‐ray has the advantages of high penetration depth, high intensity, small beam size, and energy tunability that can provide a wealth of structural and chemical information of a catalytical system. The development of synchrotron radiation sources boosts the capabilities of X‐ray and makes X‐ray‐based characterization techniques to the forefront of in situ electrochemical research. Generally, the X‐ray‐based characterization techniques can be divided into X‐ray spectroscopy, X‐ray diffraction/scattering techniques, and X‐ray imaging techniques. X‐ray spectroscopy, including XAS and X‐ray photoelectron spectroscopy (XPS), could provide elemental‐specific information of the studied sample. X‐ray diffraction/scattering techniques, including XRD and X‐ray scattering techniques (e.g., wide‐angle X‐ray scattering (WAXS) and small‐angle X‐ray scattering (SAXS)), probe the structural properties of the materials. For X‐ray imaging techniques, here we will introduce the X‐ray‐probed computed tomography (XCT) that offers direct visualization of the reaction system.

#### 2.1.1 In Situ XAS

**Figure 1:**
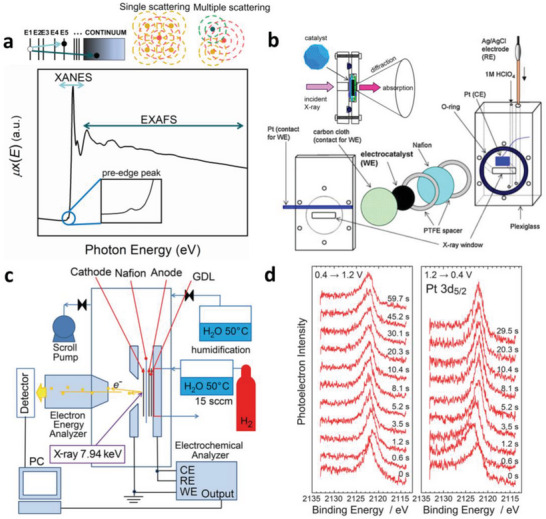
a) Schematic of XAS spectrum including the pre‐edge, XANES and EXAFS regions. The upper left is the schematic of the X‐ray absorption process and the electron excited process. Black circle: electrons. The upper right is the schematic of single and multiple scattering processes of the excited photoelectrons. The red circles indicate the absorbing atoms and the yellow and green circles indicate different neighboring atoms. The EXAFS oscillations originate from the constructive and destructive interferences between the outgoing photoelectron waves and the scattered photoelectron waves by the neighboring atom/atoms (single/multiple scattering) at the absorbing atom. b) Schematic diagram of the electrochemical cell for in situ half‐cell XAS and XRD experiment. Reproduced with permission.^[^ ^32^ ^]^ Copyright 2010, Elsevier. Inset: Reproduced with permission.^[^ ^33^ ^]^ Copyright 2016, American Chemical Society. c) Schematic of the quick near ambient pressure hard X‐ray photoelectron spectroscopy (NAP‐HAXPES) system. d) The time‐dependent in situ Pt 3d_5/2_ spectra of the Pt/C cathode catalyst acquired with the system in (c). c,d) Reproduced with permission.^[^ ^34^ ^]^ Copyright 2020, American Chemical Society.

XAS is a powerful tool for characterizing fuel cell catalysts which measures the X‐ray absorption coefficient of a material as a function of incident X‐ray photon energy. According to the incident X‐ray energy, the XAS spectrum can be roughly divided into two regions: the X‐ray absorption near edge structure (XANES) region and the extended X‐ray absorption fine structure (EXAFS) region (as shown in **Figure** **1**a). In the EXAFS region, the core electrons are excited to continuum state with the higher incident X‐ray energy. In the frame of the single/multiple scattering approach (as shown in Figure 1a), the EXAFS oscillations obtained by data reduction can be interpreted by the well‐known standard EXAFS equation expressed as the sum of a series of sine‐function‐like scattering signals from different coordination shells. With modeling and least‐squares curve fitting of the experimental data, EXAFS provides the local geometric structure including the coordination number and interatomic distance around the probed atoms. In the XANES region, the core electrons are excited to the unoccupied state and the incident X‐ray is strongly absorbed leading to a large jump, i.e., the absorption edge, in the spectrum. The edge position is sensitive to the oxidation state of the probed atom, and can be quantitatively identified using reference compounds with known oxidation states. The ligand types and the three‐dimensional coordination environment around the probed atom can also affect the XANES spectra shape and the absorption edge position. XANES contains abundant structural information, and can be applied to unveil the coordination environment, molecular orbitals, band structure, and multiple scattering.^[^ ^21^ ^]^ Proper XAS data analysis is crucial to extract structural information about the probed system. It is suggested that routinely making complementary uses of multiple XAS analysis approaches (i.e., EXAFS fitting, XANES simulation, XANES fitting, EXAFS calculation) in a global way can help identify the probed system efficiently and properly with minimized uncertainty and artificiality.^[^ ^22^ ^]^ Besides, since synchrotron radiation has become the standard X‐ray source for XAS measurement, it can provide tremendous information about electrocatalysts.

A prominent advantage of XAS is that it can be easily applied as in situ implement due to its high penetration depth and ambient operation condition. The in situ XAS study of the fuel cell can be applied either in a half‐cell environment with liquid electrolyte or a more practical full‐cell environment. Both of the two types of cells have been precisely designed for in situ XAS measurement, exhibiting their own advantages and drawbacks that make them suitable for different application scenarios.

Back in 1987, O'Grady's group^[^ ^23^ ^]^ designed an in situ electrochemical cell to obtain the X‐ray absorption spectrum of a nickel oxide electrode in alkaline electrolyte. It has been modified and applied widely for in situ half‐cell ORR research. The structure of the in situ cell is shown in Figure 1b. A carbon cloth, a studied electrocatalyst layer, a proton exchange membrane (PEM), and two polytetrafluoroethylene (PTFE) gaskets were sandwiched, and all the components were clamped tightly by the two acrylic plastic bodies with an O‐ring. Each plastic body had an X‐ray window that was glued by a thin acrylic film. A counter electrode and a reference electrode were inserted in the thicker plastic body, while a Pt ribbon on the thinner plastic body achieved electrical contact for the studied electrocatalyst was regarded as the working electrode. An electrolyte such as 1 M HClO_4_ was added to the cell. The cell is a typical three‐electrode system with liquid electrolyte that allows XAS data acquisition in both transmission and fluorescence modes while electrochemical parameters are measured simultaneously. The electrolyte in the cell keeps unchanged during the measurement, limiting the introduction of fresh electrolyte as well as variables. Based on this half‐cell, Mukerjee's group^[^ ^24^ ^]^ developed a modified flow‐through type cell with four electrolyte flow holes which allowed the introduction of fresh electrolyte without disassembling the cell during the experiment. The flow‐through cell allows the in situ research of catalyst contamination by the toxic in the electrolyte, e.g., phosphate‐anion poisoning of ORR catalyst for phosphoric acid fuel cells (PAFC),^[^ ^25^ ^]^ which is conductive to the study of poisoning mechanism.

Although the aforementioned in situ half‐cells can achieve the XAS measurement of electrocatalyst during the electrochemical reaction process, the environment in the half‐cell is quite different from that in a practical fuel cell that contains much more issues including gas pressure, mass transport, and water management, etc. Since the electrocatalysts always exhibit strikingly different performance in a real fuel cell from that in a half‐cell, the in situ half‐cells suffer from the deficiency of practical significance and could only be used as laboratory‐scale tools for the fundamental research of the catalysts. Moreover, the introduction of the liquid electrolyte might bring mobile anion contaminations such as bisulfate and sulfate.^[^ ^26^ ^]^ A practical in situ full‐cell designed on the basis of a real single fuel cell was first reported by Smotkin's group,^[^ ^27^ ^]^ providing almost the same conditions as that in a real operating fuel cell. In the in situ full‐cell, rectangular holes were milled behind the flow channel on the rear side of both end plates with only 4 mm of graphite impeding the X‐ray beam before contacting the catalyst. The chosen thickness of the X‐ray window was proved to be thin enough to make negligible contributions to the spectra and thick enough to keep the cell gas‐tight. With the in situ full‐cell, Iwasawa's group^[^ ^28^ ^]^ developed a novel time‐gating quick XAFS (TG‐QXAFS) with a millisecond time resolution, which has been widely applied to study the reaction rates, providing direct insights into real dynamic evolution of the reaction system.

However, the in situ XAS measurement in full‐cell has its own shortcomings that it cannot distinguish the signals from different layers, i.e., the two catalyst layers and the membrane layer, owing to its bulk average nature and the complex components of the cell. First, both the cathode and anode electrodes are monitored simultaneously and contribute equally to the spectra. To eliminate the interference, one can remove the catalyst in the beam window region or use catalysts without studied elements in the interference electrode. Second, the membrane on which the catalysts are coated also exhibits interference to the catalyst measurement. To avoid the interference, Jia's group^[^ ^29^ ^]^ have developed a membrane electrode assembly (MEA) fabrication method named catalyst‐coated diffusion media (CCDM) wherein the catalyst was coated onto the microporous layer rather than the membrane and the cathode CCDM was not hot‐pressed into the membrane. The method enabled a clean separation of the electrocatalyst retained in the cathode which was then transferred to a half‐cell for in situ XAS test. However, the transition of the electrode might cause destruction of the catalyst and the in situ attribute of the measurement is disputed. In the future, a quicker in situ XAS measurement in the full‐cell is needed to accurately characterize the catalyst before the contamination of membrane occurs, or an appropriate grazing incidence protocol for incident X‐ray might help to avoid the exposure of membrane.

With in situ XAS, we can obtain insights into the electronic and atomic structure evolution of catalyst and therefore help determine active sites and unravel reaction mechanisms.^[^ ^30^ ^]^ For instance, the Cu K‐edge in situ XANES spectra were collected for S‐Cu‐ISA/SNC catalyst during ORR process in alkaline media, implying Cu(+1) sites as the active sites.^[^ ^31^ ^]^ For Pt‐based ORR catalysts, the oxidation state as well as the coordination environment of Pt have been intensively probed with in situ XAS, providing descriptors of catalyst activity and durability, which will be elucidated in the following sections of this review.

#### 2.1.2 In Situ XPS

XPS is widely used to probe surface chemistry, including elemental composition and the chemical and electronic states of the target elements. XPS measurement is usually carried out in an ultra‐high vacuum which differs from the circumstance of the cathode in a fuel cell. The ambient pressure XPS (APXPS) and near‐ambient pressure XPS (NAPXPS) enable measurements under high gas pressures (<100 mbar)^[^ ^35^ ^]^ or near‐ambient pressures (for example, 0.05–0.15 mbar)^[^ ^36^ ^]^ and can be used as an in situ method.

Similar to XAS, in situ XPS could be implemented both in half‐cell and full‐cell for different research objectives. Schlögl's group^[^ ^36^ ^]^ have reported an in situ NAPXPS cell for half‐cell electrochemical research, in which the catalysts were sandwiched between a back‐wetted proton exchange membrane and a graphene layer. The graphene layer served as an electron and X‐ray transparent window and provided contact with the catalyst. Behind the membrane was the liquid electrolyte with a reference electrode and counter electrode in it. Ogasawara's group^[^ ^37^ ^]^ designed a full‐cell with two gas chambers which was compatible with in situ APXPS system. The cathode side of the MEA was exposed to the APXPS test chamber filled with oxygen gas, while the anode side was exposed to the gas chamber filled with humidified fuel gas. By connecting both electrodes to an external voltmeter or galvanometer, the XPS spectra of the cathode and the cell voltage as well as current can be recorded simultaneously.

In recent years, the application of synchrotron radiation light source, which could provide hard X‐rays of typically 6–10 keV, boosts the application of in situ APXPS. The photoelectrons excited by hard X‐rays possess larger inelastic mean free path (IMFP) which allows the measurement under higher gas pressure. Furthermore, the large IMFP in condensed matter enables the penetration of a liquid or solid overlayer which facilitates the study of complicated interfacial reactions in electrochemical systems.^[^ ^38^ ^]^ Yokoyama's group^[^ ^34^ ^]^ has designed a quick in situ NAP‐HAXPES system (as shown in Figure 1c). The cathode of the fuel cell is mounted in the measurement chamber which is filled with humidified oxygen gas and the anode is flowed with humidified hydrogen gas through a flow channel in the plate. With the system, the time‐resolved HAXPES spectrum (as shown in Figure 1d) of selected elements can be collected with a millisecond time resolution which facilitates the investigation of reaction kinetics in fuel cells.

With in situ XPS, the valence states of ORR catalyst and the nature of reaction intermediates, such as oxygen‐containing species can be characterized in real‐time which helps to infer the active ingredients during the electrocatalytic process.

#### 2.1.3 In Situ XRD

**Figure 2:**
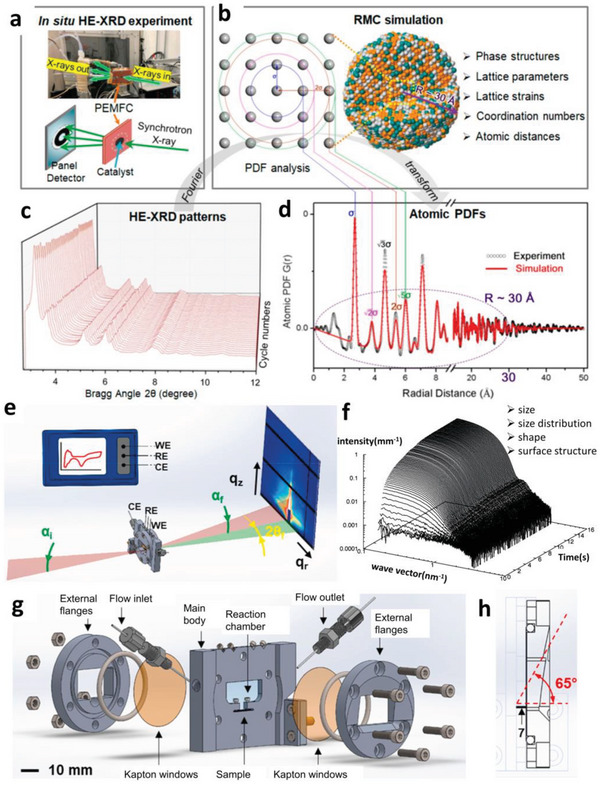
a) Setup for in situ HE‐XRD measurement of catalyst in a PEMFC device and a scheme for the detection. b) Illustration of PDF analysis and reverse Monte Carlo (RMC) simulation. c) Typical example of the HE‐XRD pattern. d) Atomic PDFs generated from experiments and simulations. a‐d) Reproduced with permission.^[^ ^40^ ^]^ Copyright 2020, American Chemical Society. e) Setup of an in situ X‐ray scattering experiment in a half‐cell. f) SAXS patterns as a function of time collected during the growth of Au NPs. Reproduced with permission.^[^ ^44^ ^]^ Copyright 2007, American Chemical Society. g) Sketch of an in situ electrochemical cell for X‐ray scattering techniques with h) the maximum exit angle designed for WAXS. e,g,h) Reproduced with permission.^[^ ^45^ ^]^ Copyright 2020, Elsevier.

XRD is widely used to determine the structural properties of crystalline or partial crystalline materials. With the assistance of theory simulations and analysis, XRD can provide abundant information including phase structure, lattice parameters, lattice strain, atomic distance, and coordination numbers. In situ XRD especially that using a synchrotron X‐ray source is an ideal structural probe for electrocatalytic mechanism research.^[^ ^39^ ^]^ High‐energy X‐ray can not only increase sample penetration that allows transmission through conventional electrochemical cells but also provide a larger range of observable reciprocal space that benefits complex structure analysis using advanced data analysis techniques such as pair distribution function (PDF), as presented in **Figure** **2**a–d.^[^ ^40^ ^]^ With the assistance of PDF technique, the application of XRD can be expanded into the analysis of materials that lack long‐range order, including glasses, gels, and liquids, which makes in situ XRD more proficient in electrocatalytic mechanism research under complicated conditions. In situ high energy XRD and PDF (HE‐XRD/PDF) has been used to probe the structural evolution of core–shell and alloy NP ORR catalysts during the ORR process to disclose the reaction mechanism and structure‐activity relationship.^[^ ^41^, ^42^ ^]^

The in situ cells for XRD experiment are similar to that for XAS, as shown in Figures 1a and 2a for the half‐cell and full‐cell, respectively. In addition, Drnec's group^[^ ^43^ ^]^ have designed an X‐ray transparent full‐cell that allowed measurement of XRD with the X‐ray beam introduced at grazing incidence, leading to a promoted signal‐to‐background ratio. The full‐cell can also be applied in the in situ X‐ray scattering methods including SAXS and WAXS, further facilitating the deconvolution of overlapping signals due to the high signal‐to‐background ratio. Furthermore, the cell was compatible with XCT techniques due to its 360^o^ transparency to X‐ray.

#### 2.1.4 In Situ X‐Ray Scattering Techniques

X‐ray scattering techniques provide structural data of both amorphous and crystalline matter at nanometer and angstrom length scales with high resolution and large detection area. X‐ray scattering techniques can be divided into WAXS and SAXS according to the incident angle of X‐ray, and both of them can be applied in situ. With WAXS, the variation of the nanostructure can be detected at atomic level, while with SAXS, the morphological changes and the intermediate structures formed during electrochemical processes can be unveiled.^[^ ^46^ ^]^ Particularly, SAXS is a powerful nondestructive tool for the characterization of nanomaterials, biomacromolecules, and polymers. It can provide the structural information including size, size distribution, shape, and surface structure in real‐time while being utilized as an in situ technique (as shown in Figure 2f).^[^ ^44^ ^]^

The design rules of in situ cells for XRD are also suitable for SAXS and WAXS, except that the materials of the X‐ray window in the cells might be different. For instance, Kapton, a material that provides low small angle scattering background, is highly suitable for the X‐ray window for SAXS. Amenitsch's group^[^ ^45^ ^]^ have reported an in situ half‐cell, similar to the flow‐through type half‐cell mentioned in Section 2.1.1 but with Kapton window for SAXS, as shown in Figure 2g. Significantly, the downstream flange was designed in the cell to allow an output angle of 65^o^ which enabled the WAXS measurement as well (as highlighted in Figure 2h).

With in situ X‐ray scattering techniques, the morphological evolution of ORR catalysts can be investigated in detail which provides deep insights into the degradation of the catalysts or the structure‐activity relationship of alloy catalysts under fuel cell operating conditions.

#### 2.1.5 In Situ XCT

During the XCT measurement, a sample is rotated around an axis perpendicular to the incident X‐ray beam, and a series of X‐ray transmission images are collected. With appropriate reconstruction algorithms, the collected images are computed and 3D reconstructed images are obtained.^[^ ^47^ ^]^ Over the past decades, with the superior synchrotron X‐ray, state‐with‐art detectors, and progressive tomographic data collection methods, in situ XCT techniques are well developed to enable the measurement of fuel cell filled with water and gases, which provides an opportunity to monitor the morphological evolution inside a PEMFC. The tomographic nature of the technique enables the layer‐by‐layer detection thus eliminating the interferences among different components.

**Figure 3:**
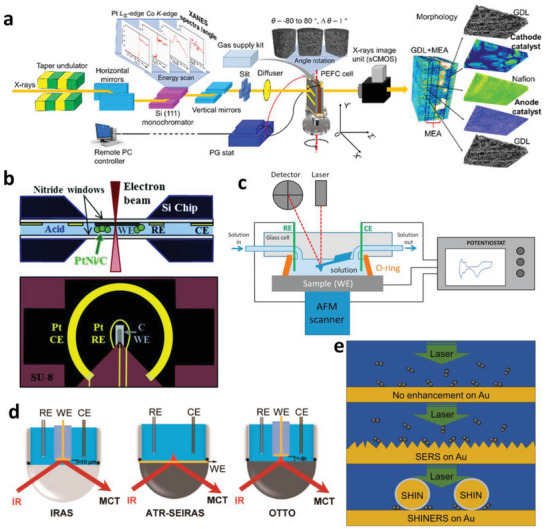
a) Schematic of a typical in situ CT‐XANES measurement of a PEMFC. Reproduced with permission.^[^ ^50^ ^]^ Copyright 2019, American Chemical Society. b) Overview of in situ electrochemical TEM cell setup, with a cross‐section of the liquid cell holder on the top and view of the electrodes on the bottom. Reproduced with permission.^[^ ^51^ ^]^ Copyright 2019, the Royal Society of Chemistry. c) Schematic illustration of an in situ electrochemical atomic force microscopy (EC‐AFM). Reproduced with permission.^[^ ^53^ ^]^ Copyright 2017, Elsevier. d) Diagrams of IR reflection‐absorption spectroscopy (IRAS or IRRAS), attenuated total reflectance‐surface‐enhanced IR absorption spectroscopy (ATR‐SEIRAS), and Otto‐ATR‐IR. Reproduced with permission.^[^ ^55^ ^]^ Copyright 2022, American Chemical Society. e) Differences among in situ Raman spectra on smooth (upper), roughened (middle), and SHIN's drop cast (lower) on the surface of gold. Adapted with permission.^[^ ^56^ ^]^ Copyright 2016, American Chemical Society.

Furthermore, combining the tomographic techniques with XANES and XRD, named in situ CT‐XANES^[^ ^48^ ^]^ and CT‐XRD,^[^ ^49^ ^]^ CT techniques with chemical discrimination are afforded, which are of more practical significance. The scheme of an in situ CT‐XANES measurement of a PEMFC is displayed in **Figure** **3**a,^[^ ^50^ ^]^ revealing the evolution of morphology as well as the Pt valence and density during the ADTs.

### 2.2 Other In Situ Imaging Techniques

Besides XCT, there are two imaging methods, TEM and AFM, that are more commonly used in the in situ study of ORR. Different from XCT techniques that could visualize a full‐cell, in situ TEM and AFM can only provide information of electrocatalysts in half‐cell.

The most commonly used in situ imaging technique is in situ liquid cell TEM (LC‐TEM). The in situ LC‐TEM measurement is applied in dedicated in situ TEM holders. The schematic of a commercial in situ LC‐TEM holder is shown in Figure 3b,^[^ ^51^ ^]^ composed of a three‐electrode flow cell with a silicon nitride electron beamline window. In situ LC‐TEM allows tracking the nanoscale changes of the catalyst under electrochemical conditions, but the results are of low resolution due to the interference of the liquid electrolyte. It is a challenge to make the electrolyte layer thinner to reduce the interference while maintaining the electrochemical environment in the cell for the future development of in situ LC‐TEM holders.

The so‐called identical‐location TEM (IL‐TEM), a quasi in situ technique, is widely used to track the morphological evolution of electrocatalysts due to its higher resolution than LC‐TEM.^[^ ^52^ ^]^ A TEM grid is used as the working electrode that experiences electrochemical processes in a liquid cell and the morphology of an identical region of the electrode surface before and after the processes is detected ex situ with TEM. The TEM measurement is performed in a vacuum with dried samples, so that analytical scanning transmission electron microscopy (STEM) methods such as energy‐dispersive X‐ray spectroscopy (EDS) and electron energy loss spectroscopy (EELS) can be performed and provide more information as compared to LC‐TEM.

AFM is one of the most important methods for obtaining surface structural information, which has been developed as EC‐AFM to be applied in in‐situ electrochemical research. The scheme of an EC‐AFM is presented in Figure 3c. The sample is mounted on the AFM head, into an electrochemical flow‐through liquid cell. The liquid cell contains a three‐electrode system where the sample is used as the working electrode.^[^ ^53^ ^]^ EC‐AFM uses tapping‐mode to gain high scanning speed, and enables the observation of surface electrochemical reactions in real‐time. For ORR research, the Pt‐based particle degradation, for instance Pt NP dissolution, can be directly observed with EC‐AFM.^[^ ^54^ ^]^

### 2.3 In Situ Vibrational Spectroscopy

The detection and monitoring of intermediates and products during reaction process are of great importance for reaction mechanism research, especially for the ORR process which involves various O‐containing intermediates. One of the most effective in situ characterization methods to monitor the O‐containing species is vibrational spectroscopy, including electrochemical IR and Raman spectroscopy.

IR spectroscopy is the absorption of IR radiation by molecules when the frequency of IR radiation is equal to the vibrational modes of the molecules with a changing dipole moment.^[^ ^57^ ^]^ In situ electrochemical infrared spectroscopy (EIRS) was first reported in 1980 by Kunimatsu's group^[^ ^58^ ^]^ and then developed to provide information on the dynamic process and key intermediates, which has played a vital role in understanding the mechanism of the electrochemical reaction. Besides the less used transmission mode, there are three reflection‐mode in situ EIRS methods, that is, the external IRAS (or IRRAS), attenuated total reflection absorption IR spectroscopy in Kretschmann configuration (known as ATR‐IR or ATR‐SEIRAS) and that in Otto configuration (Otto‐ATR‐IR). The schematic diagrams of the three IR methods are shown in Figure 3d.^[^ ^55^ ^]^ The ATR‐SEIRAS possesses high surface sensitivity while the other two methods are more suitable for detecting species in the electrolyte. To the best our knowledge, in situ IR measurements are mostly applied in half‐cell environment with the existence of liquid electrolyte. The application in a real fuel cell has not been reported yet, which might owe to the weak IR signals and the abundant interferences in a full‐cell.

As a complementary method to IR, Raman spectroscopy measures the bond vibrations that induce changes in the polarizability of molecules. Raman spectroscopy is based on the inelastic scattering of photons while interacting with molecular vibrations. Due to the low probability of Raman scattering, the spectroscopic signal of Raman is often weak and some enhanced Raman techniques such as surface enhanced Raman scattering (SERS) have been developed.^[^ ^59^ ^]^ SERS is a powerful fingerprint vibrational spectroscopy that can be used for in situ monitoring and identification of trace species with single‐molecule sensitivity.^[^ ^60^ ^]^ However, SERS is mostly used on Au, Ag, and Cu metal surfaces that have intrinsic ability to amplify Raman signals while the other metals, such as Pt has been rarely investigated because of the low Raman enhancement. Tian's group^[^ ^61^ ^]^ have developed a practical surface vibrational spectroscopy named shell‐isolated NP‐enhanced Raman spectroscopy (SHINERS) which makes it possible to obtain enhanced Raman signals from any kind of substrate thus extending its application in electrochemical investigation. In SHINERS method, an ultrathin and uniform silica shell is coated onto a gold NP which can efficiently enhance the Raman signal of molecules located near the NP surface. Different core (Au and Ag) and shell (SiO_2_ and Al_2_O_3_) materials are used as SHINs with various shapes to enhance the SERS activity. The SHINs are dispersed on the surface of an electrode where the Raman spectra are collected during the electrochemical processes. The schemes of Raman, SERS and SHINERS are presented in Figure 3e,^[^ ^56^ ^]^ exhibiting the different implement of the methods.

Although IR and Raman are both vibrational spectroscopies, the principles of them are distinct, which makes the different applications of the two techniques. For instance, IR is sensitive to water and could probe interfacial water and adsorbed electrolyte in electrochemical reaction,^[^ ^62^ ^]^ while Raman has no interference of water and could provide information of bond vibrations in low frequency range.^[^ ^63^ ^]^

### 2.4 Theoretical Calculations

Theoretical calculation is an important tool for the investigation of electrocatalytic mechanism which can elucidate the charge distribution, adsorption energies, electronic structure, and defect states of the species during the reactions.^[^ ^64^, ^65^ ^]^ With calculation methods, the catalytic process can be simulated at atomic level which can explain some experimental phenomena and make some predictions about the activity of catalysts. For ORR, Nørskov and coworkers^[^ ^66^, ^67^ ^]^ have done abundant calculations to investigate the correlations between the binding energy of O‐containing species on catalyst surface and the activity of the catalyst as well as the surface d‐band center and the activity. Based on the calculation results, they explained why Pt is the most active metal for ORR and predicted that by alloying a second metal, the electronic structure of Pt can be tuned to generate better ORR activity, which was confirmed by experiments (which will be discussed in detail in Section 4.1.1).

By combining theoretical calculations with in situ characterization methods, the reaction mechanism can be explored with high proficiency. For instance, the d‐band position of catalyst, one of the ORR activity descriptors, can be obtained experimentally with in situ techniques such as XAS. With the assistance of theoretical calculation, the change of d‐band position can be explained more in‐depth. On the other hand, theoretical calculations can assist in analyzing some in situ spectroscopy. For instance, density functional theory (DFT) methods can be employed to calculate the vibrational frequencies of target species and help assign the in situ Raman and IR peaks.^[^ ^68^, ^69^ ^]^

In general, the in situ techniques introduced above could provide an opportunity to capture the real picture of ORR processes. In situ XAS provides the local structural and electronic information of the reaction system. With the assistance of in situ XRD and X‐ray scattering techniques, the long‐ and nano‐range structural evolution of the catalysts could be monitored. The properties of the intermediate species could be tracked with in situ XPS, IR, and Raman. The degradation of ORR catalysts during the electrochemical processes could be directly observed with in situ imaging techniques.

The implement of these in situ methods relies heavily on the design of in situ cells. As overviewed above, the half‐cell enables in situ study of catalysts under a certain condition and is more adaptive with the characterization techniques due to its simple components. The in situ full‐cell is almost consistent with the practical fuel cell, exhibiting the minimum gap between the realistic electrocatalytic reaction conditions and the measurement conditions. Nevertheless, the in situ study in a full‐cell prompts severe demands for the detection methods, such as deep penetration and high resolution, due to the complicated circumstances in the operating cell, which hinders the application of it.

Besides the aforementioned methods, some advanced techniques including scanning tunneling microscopy (STM),^[^ ^70^, ^71^ ^]^ Mössbauer spectroscopy,^[^ ^72^, ^73^ ^]^ etc., can also be applied as in situ methods for electrochemical research. However, the application of these techniques is limited by their inherent attributes. For instance, although STM could monitor the structural evolution and adsorption/desorption of reactants with atomic resolution, the rigorous requirement for smooth surface limits its application in the in situ study of heterogeneous catalysis.

## 3 In Situ Study of the ORR Mechanism and the Failure Mechanisms of ORR Catalyst

### 3.1 The ORR Mechanism

As one of the fundamental reactions in electrocatalysis, the mechanism and kinetics of ORR have been extensively investigated.^[^ ^74^, ^75^, ^76^ ^]^ General ORR mechanisms in acidic media are considered to occur through two main pathways: a four‐electron pathway through which the O_2_ is directly reduced to H_2_O; an indirect two‐electron reduction pathway in which O_2_ is reduced to H_2_O_2_, followed by a two‐electron transfer reduction of H_2_O_2_ to H_2_O. Due to the complicated reaction conditions, both pathways often occur concomitantly as competing reactions which concerns the selectivity of ORR catalysts. Herein, the ORR mechanisms on Pt‐based catalysts through the four‐electron pathway are discussed with the in situ study of three crucial reaction intermediates, O_2_* (asterisk (*) indicates species adsorbed on the surface), HO_2_*, and *OH, on Pt crystalline surface, NPs, and commercial Pt/C catalysts.

**Figure 4:**
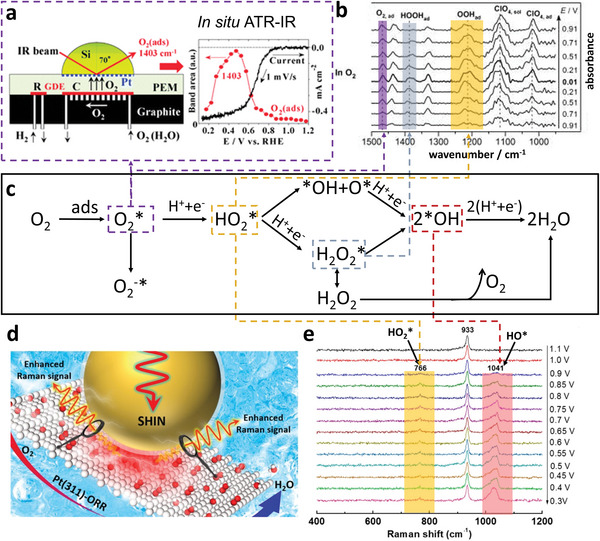
a) In situ ATR‐IR observation of O_2_* on Pt NPs. Reproduced with permission.^[^ ^77^ ^]^ Copyright 2010, the Royal Society of Chemistry. b) In situ ATR‐IR spectra of Pt/C during ORR in 0.1 M HClO_4_ solution. The band at 1212 ± 3 cm^−1^ was assigned to the O–O stretching mode of OOH_ad_(HO_2_*); the band at 1386 ± 4 cm^−1^ was assigned to the OOH bending mode of HOOH_ad_(H_2_O_2_*); the band at 1468 cm^−1^ was assigned to the O–O stretching mode of weakly adsorbed O_2,ad_(O_2_*). Reproduced with permission.^[^ ^78^ ^]^ Copyright 2018, Wiley‐VCH. c) ORR mechanisms in acidic media (with O_2_ adsorbed in associative pathway). d) Schematic of electrochemical SHINERS (EC‐SHINERS) study of the ORR on Pt surface. e) EC‐SHINERS spectra of the ORR system on Pt(311) surface in HClO_4_ solution saturated with O_2_ under different potentials. d,e) Reproduced with permission.^[^ ^79^ ^]^ Copyright 2020, American Chemical Society.

The first step of ORR is the adsorption of O_2_ on Pt surface through associative or dissociative pathways. Since the associative pathway dominates at 0.8 V and below,^[^ ^66^ ^]^ the operation potential in the cathode of a PEMFC, the ORR processes starting with associative adsorption of O_2_ are focused in this section. Watanabe's group^[^ ^77^ ^]^ have directly observed O_2_* on Pt NPs with in situ ATR‐IR during the ORR process, verifying the associative adsorption of O_2_. The infrared band at 1400–1403 cm^−1^ in **Figure** **4**a was assigned to the O–O vibration of O_2_* due to its absence in the N_2_ atmosphere and insensitivity to the change from H_2_O to D_2_O humidification. The existence of the band at the high potential up to 1.1 V excluded the possibility of O_2_^−^*. Vincent's group^[^ ^78^ ^]^ further confirmed the associative adsorption of O_2_ on commercial Pt/C catalyst in O_2_‐saturated HClO_4_ solution with in situ ATR‐IR. The results disclosed the existence of O_2_*, HO_2_*, and H_2_O_2_* during the ORR process, as shown in Figure 4b, which strongly verified the mechanism depicted in Figure 4c.

The associatively adsorbed O_2_* then experiences a proton‐coupled electron transfer (PCET) process, generating HO_2_* (as depicted in Figure 4c) which is regarded as the central ORR intermediate in acidic media since its transformation direction determines whether the whole ORR proceeds through the four‐electron or two‐electron pathway. With the assistance of in situ EC‐SHINERS and DFT calculations, Li's group^[^ ^69^, ^79^ ^]^ confirmed the existence of HO_2_* on the surface of Pt(111), Pt(211), and Pt(311) electrode during the ORR process (as depicted in Figure 4d,e). The Raman peaks around 766 cm^−1^ were assigned to the O–O stretching vibration of the bridged adsorbed HO_2_*. They have also demonstrated that the binding energy of HO_2_* on different surfaces could reflect the catalytic activity of the surfaces. For instance, the adsorption energy of HO_2_* on Pt(311) is higher than that on Pt(211), which hinders the diffusion and transformation of HO_2_*, leading to a weaker ORR activity of Pt(311).

The subsequent reactions of HO_2_* split at a bifurcation point: One is a chemical process to *OH and O* followed by the conversion of O* to *OH; the other is a PCET to H_2_O_2_* followed by the O–O breaking process to form two *OH. *OH is the key intermediate of ORR that Pt–OH bond strength is a critical descriptor of catalyst activity.^[^ ^66^ ^]^ Probing the adsorption behavior of *OH on different Pt surfaces helps evaluate the activity of the surfaces and thus provides efficient guidelines for the rational design of highly active ORR catalysts through shape control strategies. Li's group^[^ ^69^ ^]^ have investigated the intermediates during ORR on Pt(111), Pt(110), and Pt(100) electrode with in situ EC‐SHINERS. The presence of *OH on Pt(110) and Pt(100) indicated the strong Pt–OH binding energy, leading to a weaker ORR catalytic activity of the two surfaces, while the absence of *OH on Pt(111) indicated a quick transformation of *OH to H_2_O owing to the high catalytic activity of Pt(111). The results accounted well for the fact that Pt NPs in octahedral shape with enriched (111) facet exhibits higher ORR activity. Besides the Pt surface, Ogasawara's group^[^ ^37^ ^]^ realized the detection of *OH on Pt/C catalyst in a full fuel cell during the ORR process. Two phases of *OH, hydrated *OH and non‐hydrated *OH, coexisted on the Pt/C catalyst under operating conditions which was confirmed by a combination of in situ APXPS and DFT calculation. The hydrated *OH was confirmed to possess lower reactivity in comparison with non‐hydrated *OH, providing a new insight of improving the ORR catalytic activity by manipulating the hydrophobic and hydrophilic properties of the catalysts.

In general, the in situ probing of the intermediates is of significant importance which could not only help elucidate the reaction mechanisms but also provide descriptors of the catalytic activity (i.e., the binding energy of the intermediates) and consequent efficient guidelines for the rational design of high‐performance catalysts. However, only the abundant intermediates with long lifetimes could be detected directly with in situ techniques. As for the species with low concentrations and short lifetimes, some strategies could be taken to make it enriched and stable for detection. For instance, Feliu's group^[^ ^80^ ^]^ used NaF buffer to adjust the pH of HClO_4_ electrolyte. When the pH was above the pK_a_ of O_2_H·/O_2_^−^, the lifetime of O_2_^−^ increased and O_2_^−^* originating from O_2_*(as shown in Figure 4a) would accumulate at the Pt surface and therefore can be detected with in situ IRRAS.

### 3.2 Catalyst Failure Mechanisms

#### 3.2.1 Observation and Decoupling of Pt NP Degradation Processes

**Figure 5:**
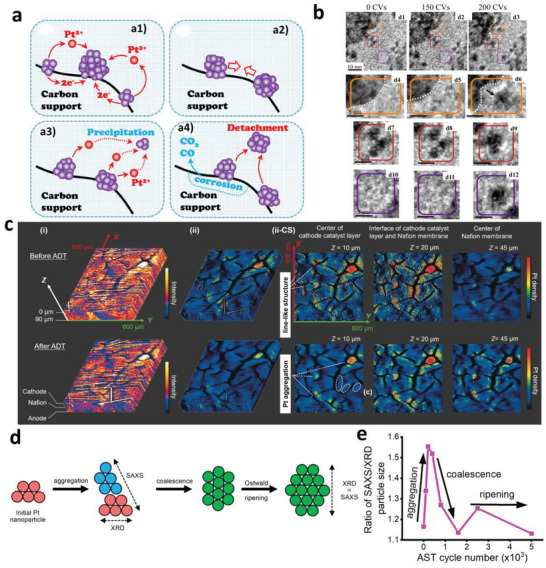
a) Degradation mechanisms for Pt NPs: Ostwald ripening (a1), coalescence or aggregation (a2), migration (a3, including dissolution, diffusion, and precipitation processes), and detachment (a4). Reproduced with permission.^[^ ^7^ ^]^ Copyright 2020, Elsevier. b) Direct observation of catalyst degradation with in situ LC‐TEM: (d4−d6) detachment (orange squares), (d7−d9) coalescence (garnet squares), and (d10−d12) precipitation within the electrolyte (purple squares). Reproduced with permission.^[^ ^81^ ^]^ Copyright 2020, American Chemical Society. c) 3D images of a PEMFC acquired with in situ CT‐XANES before and after 20000 ADT cycles. i) 3D images of morphology. ii) 3D images of the Pt density. ii‐CS) the cross‐sectional images of ii) at Z = 10, 20, and 45 µm. Reproduced with permission.^[^ ^48^ ^]^ Copyright 2017, Wiley‐VCH. d) Structural evolution of the electrocatalyst during ADTs, showing how SAXS and XRD probe distinct but complementary aspects of the nanomorphology. e) Ratio of the SAXS particle size to XRD crystalline size in the cathode over the ADTs. d,e) Reproduced with permission.^[^ ^83^ ^]^ Copyright 2022, Elsevier.

During the automotive operation of PEMFCs, the degradation processes of Pt NPs in Pt/C catalyst including Ostwald ripening, aggregation/coalescence, migration, and detachment due to the carbon corrosion (as presented in **Figure** **5**a) are inevitable, leading to the decrease of the active sites of catalysts and consequent performance decay of the cell.

With in situ imaging techniques, the degradation of Pt NPs can be observed in real‐time both in half‐cell and MEA. Rizza's group^[^ ^81^ ^]^ have observed the detachment, coalescence, dissolution, migration and precipitation of Pt NPs (as presented in Figure 5b) of a commercial Pt/C catalyst during cyclic voltammetry (CV) with in situ LC‐TEM. However, the carbon corrosion was not presented due to the low resolution of LC‐TEM. Maillard's group^[^ ^82^ ^]^ have applied analogous experiments with IL‐TEM that provided images with higher resolution and revealed the process of carbon corrosion. An in situ CT‐XANES measurement^[^ ^48^ ^]^ revealed the morphology, Pt density and valence evolution in different depths of an MEA (as presented in Figure 5c) during ADTs, including the Pt NP degradation in the catalyst layer, the migration of Pt from catalyst layer to membrane layer, and the oxidation of the catalyst surface under high potentials.

Significantly, the degradation processes of Pt NPs always occur concomitantly during operation but the imaging techniques are incapable of distinguishing them. However, the decoupling of those processes is meaningful which can help tease apart the mechanisms and develop efficient mitigation strategies correspondingly. Drnec's group have proposed some strategies to decouple the degradation processes with the combination of in situ structural probing techniques as well as electrochemical methods. It is recognized that the growth of Pt NPs at the cathode occurs frequently during the operation of PEMFCs. Processes including aggregation, Ostwald ripening, and coalescence can all induce the NP growth. Drnec's group^[^ ^83^ ^]^ found that a simultaneous in situ XRD and SAXS measurement allows the decoupling of aggregation, coalescence, and ripening of Pt NPs. XRD and SAXS are both sensitive to the particle size distribution of the catalyst. However, SAXS measures the overall shape and size of a NP, while XRD is only sensitive to the size of an individual crystalline (as depicted in Figure 5d). The polycrystallinity and the aggregation of NPs can therefore be distinguished with the difference of SAXS and XRD sizes. Furthermore, grain boundaries between aggregated Pt NPs create local distortion of the crystal lattice, named “microstrain” that could induce extra peak broadening in XRD patterns which can be deconvoluted in the Rietveld analysis. Nevertheless, unalloyed, well‐dispersed Pt/C exhibits no microstrain broadening. Thus, the detection of microstrain broadening serves as an independent measurement of the aggregation. Following those rules, the mechanisms of Pt NP growth during the ADT cycles were unambiguously revealed, as shown in Figure 5e. At the first 400–500 ADT cycles, the SAXS/XRD ratio of the cathode Pt increased due to the particle aggregation. The microstrain broadening can also be detected between 100–400 ADT cycles and was undetectable before and after this stage. After this stage, the SAXS/XRD ratio decreased, indicating the coalescence of the aggregated Pt NPs. Further, the ratio tended to be constant, indicating a switch of the dominant mechanism to Ostwald ripening. Moreover, they also suggested that the ratio of the electrochemical active surface area (ECSA) to the quantity of Pt obtained with in situ XRD is an efficient descriptor to probe the degradation mechanisms.^[^ ^84^ ^]^ Processes such as partial corrosion produce increasing ECSA/Pt loading ratios. Aggregation and ripening degradation produce decreasing ratios. Catalyst detachment from the carbon support would maintain the ratios.

#### 3.2.2 The Oxidation of Pt

**Figure 6:**
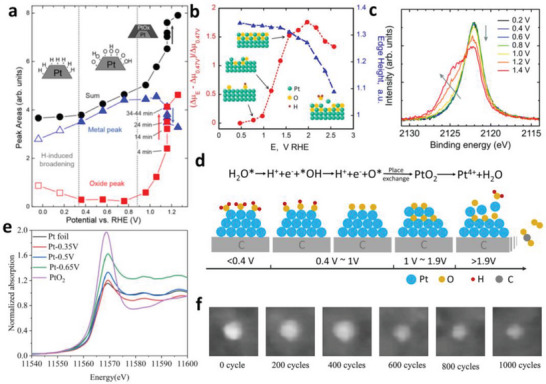
a) Areas of the fitted components for the full set of in situ XANES spectra acquired for Pt on glassy carbon, as well as their sum, plotted as a function of the sample potential. Reproduced with permission.^[^ ^86^ ^]^ Copyright 2012, American Chemical Society. b) Changes in white line intensity (red curve) and edge height (blue curve) as a function of the applied potentials observed in in situ XANES of the Pt/C catalyst. Also shown are schematics of the process of Pt oxide formation and its dissolution. Reproduced with permission.^[^ ^33^ ^]^ Copyright 2016, American Chemical Society. c) In situ NAP‐HAXPES Pt 3d_5/2_ spectra of the Pt/C catalyst at the cathode under various cell voltages. The shoulder peak indicates the existence of Pt(I) and Pt(II) species. Reproduced with permission.^[^ ^92^ ^]^ Copyright 2017, the Royal Society of Chemistry. d) The structural evolution of Pt/C catalyst under different potentials. e) XANES spectra collected at Pt L_3_ edge of Pt/C at different potentials. Reproduced with permission.^[^ ^93^ ^]^ Copyright 2022, Elsevier. f) High‐speed AFM images of cubic Pt NP (imaged area: 70 nm × 70 nm) in 0.1 M HClO_4_ saturated air during potential cycles between 0.05 and 1.6 V (vs RHE). Reproduced with permission.^[^ ^54^ ^]^ Copyright 2016, Elsevier.

For commercial applications of PEMFC, the automotive operations, including open circuit/idling and startup‐shutdown, are common and would bring about large interfacial potentials up to about 1.4–1.6 V (vs RHE) at the cathode.^[^ ^85^ ^]^ The electrochemical oxidation of Pt surface through water dissociation would occur under such high potentials. To eliminate the interference of O_2_, the oxidation process has been studied under N_2_ atmosphere by probing Pt surface state under raising potentials. Based on the results of an in situ XAS measurement, Roldan Cuenya's group^[^ ^86^ ^]^ concluded that the surface states of Pt NPs on glassy carbon in HClO_4_ solution can be divided into three potential regimes: hydrogen chemisorption on Pt between 0 and ≈0.3 V, oxygen chemisorption (OH or O) on Pt between 0.3 and 0.96 V, and Pt oxide formation at or above 0.96 V (as shown in **Figure** **6**a). In addition to Pt NPs, the oxidation of Pt on crystalline surface and in Pt/C catalysts contains the similar steps, during which the intermediates, the surface state and structural evolution of Pt have been precisely investigated with in situ techniques.

Nakamura's group^[^ ^87^ ^]^ studied the oxidation of Pt(111), Pt(110), and Pt(100) surfaces in HF with in situ IRAS, which confirmed the existence of *OH on Pt surfaces in acidic media in a certain potential window. The *OH is originated from the adsorption of H_2_O on the Pt surface via the following schemes:

(3) $$H_{2}O*\to *\mathrm{OH}+H^{+}+e^{-}$$

(4) $$*\mathrm{OH}\to O*+H^{+}+e^{-}$$

Equation (3) is regarded as the first step of Pt surface oxidation through water dissociation which was confirmed by DFT calculation^[^ ^88^ ^]^ and an ex situ XPS measurement.^[^ ^89^ ^]^ It is worth noting that the *OH discussed here should be differentiated from that in the ORR mechanism section. This *OH originates from the water while the latter originates from O_2_. The similarity is that *OH here is a key intermediate in the oxidation process of Pt, drawing forth strategies for promoting the anti‐oxidation activity by manipulating the adsorption and generation of *OH, which will be discussed in detail in Section 4.2.1.

At higher potentials, the *OH is further oxidized to O* through the pathway demonstrated in Equation (4). With the assistance of in situ time‐resolved XAS and XRD, Imai's group^[^ ^90^ ^]^ have substantiated the transformation of *OH to O* by attentively analyzing the bond length of Pt–O and Pt–Pt species during the oxidation process of a commercial Pt/C catalyst in HClO_4_ solution. The Pt–O bond formed just after the oxidation was assigned to Pt–OHH (adsorbed water molecule) and/or Pt–OH with a bond length of 2.2–2.3 Å. Then the Pt–O bond length reduced to 2.0 Å, indicating the discharge of *OH to O*.

The O* then performs a place‐exchange process with Pt atoms, producing bulk Pt oxide under higher potentials. Koper's group^[^ ^91^ ^]^ have observed the PtO_x_ (mainly *α*‐PtO_2_) species on Pt(111) and Pt(100) surfaces in HClO_4_ under potentials beyond 1.3 V with the in situ SHINERS experiments. For commercial Pt/C catalyst in acid electrolyte, Adzic's group^[^ ^33^ ^]^ have revealed the place‐exchange process at potentials up to 1.5 V with a combined in situ XANES and in situ XRD measurements (as shown in Figure 6b). While in a full fuel cell, the generation of Pt oxide on the surface of Pt/C catalyst was confirmed to start under potentials higher than 1.0 V, as demonstrated by Yokoyama's group^[^ ^92^ ^]^ with in situ NAP‐HAXPES (as presented in Figure 6c). It is obvious that the starting potential of place‐exchange process changes under different conditions. However, according to the in situ studies mentioned above, the generation of Pt oxide could be avoided to keep the operation potential under 1.0 V.

At potentials higher than 1.9 V (around the onset potential of the oxygen evolution reaction (OER) of Pt NPs), some irreversible degradation processes would occur and lead to permanent damages to the catalyst. The white line intensity and the edge height of the spectra both decreased in the Pt L_3_ edge in situ XANES spectra collected on a Pt/C catalyst in acid electrolyte (Figure 6b), indicating the dissolution of PtO_2_ into the electrolyte (Equation 5) under more acidic environment near the surface caused by the OER (Equation 6). Besides, carbon corrosion takes place (Equation 7) under such high potentials, and further induces the detachment of Pt NPs.

(5) $${\mathrm{PtO}}_{2}+4H^{+}\to {\mathrm{Pt}}^{4+}+2H_{2}O$$

(6) $$2H_{2}O\to O_{2}+4H^{+}+4e^{-}$$

(7) $$2C+2H_{2}O\to 2{\mathrm{CO}}_{2}+4H^{+}+4e^{-}$$

According to the mechanisms demonstrated above, the complete electrochemical oxidation process of Pt/C without O_2_ under increasing potentials could be divided into four steps (as shown in Figure 6d): 1) Water adsorption under potentials below 0.4 V; 2) Generation of *OH and the following discharging to O* between 0.4–1.0 V; 3) Place‐exchange of O* with Pt (generation of Pt oxide) beyond 1.0 V; 4) Dissolution of Pt oxide, carbon corrosion, and OER beyond 1.9 V.

It should be noted that during the practical operation of PEMFCs, there is always O_2_ existing in the cathode and the in situ study of Pt oxidation with the presence of O_2_ is of more practical significance. Liu's group^[^ ^93^ ^]^ have monitored the oxidation of Pt in a commercial Pt/C catalyst under air with the in situ XANES spectra collected at Pt L_3_ edge (as shown in Figure 6e). The intensity of white lines which is related to the 5d‐electron vacancy of Pt increased with the rise of potential, indicating that the degree of Pt oxidation became higher. The following dissolution of Pt oxides has also been confirmed with in situ high speed AFM (as shown in Figure 6f), as depicted by Mizumoto's group.^[^ ^54^ ^]^ The dissolution of cubic Pt NPs started below the potential where most of the Pt oxides were reduced, indicating that the dissolution was induced by the reduction of Pt oxides. Tada's group^[^ ^94^ ^]^ have contrasted the Pt oxidation processes with and without the presence of O_2_ in the cathode of a practical MEA with in situ time‐resolved QXAFS. The results suggested that with the presence of O_2_, the oxidation of Pt proceeded with the competition of O_2_ and H_2_O, leading to a faster charging rate of Pt. However, the rates of Pt–O formation and Pt–Pt breaking (i.e., the generation of Pt oxide) were dominated by the cell voltage and has no concern with the atmosphere with/without O_2_. Thus, the aforementioned Pt oxidation mechanism without O_2_ remains valid for the oxidation with O_2_, which reflects the real circumstances of a PEMFC.

#### 3.2.3 The Poisoning of Pt Catalysts

The poisoning of Pt by the contaminants in the feeds during fuel cell operation plays an important role in the deterioration of ORR catalysts. The main reason of the poisoning is the blockage of the active sites on Pt surface, which could be quantified with the decreased integrated area in the hydrogen adsorption/desorption region in CV curves. However, electrochemical methods cannot provide detailed information of the poisoning process. The application of in situ techniques unveils the adsorption behavior of the contaminants, including the adsorption configuration, dissociation, and removal process, providing profound insights into the poisoning mechanisms and thus efficient guidance for the mitigation strategies.

**Figure 7:**
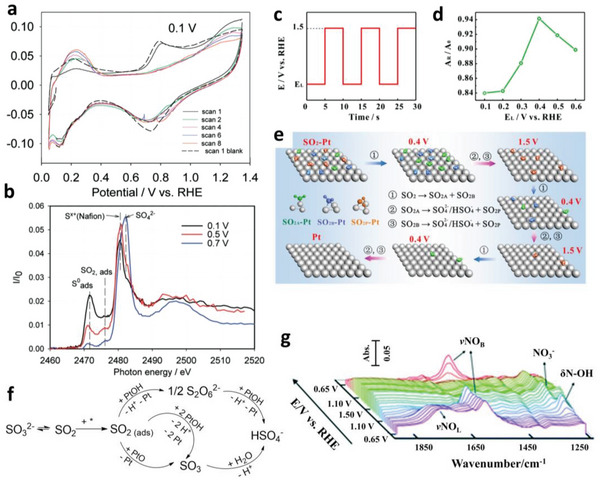
a) CV curves recorded after exposure of the Pt/C electrode held at 0.1 V to 1000 ppm SO_2_ in N_2_. b) Pre‐edge background corrected XANES spectra measured after carrying out the exposure experiment depicted in a) while holding the working electrode at 0.1 (black), 0.5 (red), and 0.7 (blue) V. a,b) Reproduced with permission.^[^ ^96^ ^]^ Copyright 2011, American Chemical Society. c) The square wave‐based protocol to regenerate the SO_2_‐poisoned Pt surface. d) Curve of A_R_/A_0_ as a function of E_L_. A_R_ is the ECSA of the regenerated Pt/C catalyst and A_0_ is the ECSA of clean Pt/C; and e) illustration for the regeneration of Pt poisoned by SO_2_. c‐e) Reproduced with permission.^[^ ^97^ ^]^ Copyright 2021, American Chemical Society. f) SO_2_ oxidation mechanism on Pt electrocatalyst. Reproduced with permission.^[^ ^98^ ^]^ Copyright 2019, American Chemical Society. g) In situ ATR‐SEIRA spectra of a NO‐poisoned polycrystalline Pt electrode in an Ar‐saturated 0.1 M HClO_4_ solution during potential sweep. Reproduced with permission.^[^ ^99^ ^]^ Copyright 2022, the Royal Society of Chemistry.

SO_2_ is a common air impurity that can severely poison the Pt catalyst that trace amounts of SO_2_ can cause significant loss of ORR performance. For instance, 1 ppm SO_2_ in the air would cause a 35% drop of the cell voltage after 100 hours of operation.^[^ ^95^ ^]^ Northrup's group^[^ ^96^ ^]^ have investigated the adsorption of SO_2_ on Pt/C catalyst coated membrane under different electrode potentials using a combination of in situ sulfur K‐edge XANES and electrochemical techniques. The suppression of the hydrogen adsorption/desorption region in the CV curves (as shown in **Figure** **7**a) collected after the electrode exposure to SO_2_ indicated the occupation of Pt active sites. The in situ XANES spectra represented the corresponding adsorbates, sulfur (S^0^) and SO_2_, under potentials below 0.7 V (as shown in Figure 7b).

Based on the in situ XANES results, the adsorbed S species were oxidized to (bi)sulfate ions under potentials higher than 0.7 V. The CV curves showed that no suppression of the hydrogen region (i.e., no poisoning of Pt surface) was observed after exposure of the electrode to SO_2_ at 0.9 V. Thus, the oxidation of adsorbed sulfur species at potentials beyond 0.9 V could be carried out through a triangle potential wave, which is generally used in the literature, to remove the sulfur and restore the cell performance. Furthermore, with the assistance of in situ ATR‐SEIRAS and DFT calculations, a more efficient square wave‐based regeneration protocol (as shown in Figure 7c) has been proposed by Yin and coworkers.^[^ ^97^ ^]^ They suggested that parallel‐bonded SO_2_ cannot be electro‐oxidized at a high potential but can be converted to more easily oxidized atop‐bonded and bridge‐bonded SO_2_ at a low potential, named E_L_. With a best E_L_ (determined with the A_R_/A_0_‐E_L_ curve as shown in Figure 7d), the square‐based wave could fulfill the conversion and completely regenerate the catalyst within only three cycles, as depicted in Figure 7e.

To further investigate the SO_2_ oxidation reaction (SO_2_OR), Córdoba de Torresi's group^[^ ^98^ ^]^ have conducted an in situ IRRAS measurement of SO_2_OR on Pt electrode. As shown in Figure 7f, dithionate (S_2_O_6_^2−^) was confirmed as a reaction intermediate of SO_2_OR and Pt–OH were suggested as oxidative species in the acidic media. Herein, *OH emerged again either from O_2_ or H_2_O, indicating that the strategies of manipulating *OH for promoting ORR activity or developing anti‐oxidation catalysts might play a part in developing SO_2_‐resistant catalyst.

NO_x_ is another kind of common air contaminants that can deactivate the ORR catalysts. Yin's group^[^ ^99^ ^]^ have investigated the adsorption behavior of NO on a polycrystalline Pt electrode in HClO_4_ solution during the potential sweep with in situ ATR‐SEIRAS. The in situ research suggested that the adsorbed NO could be removed through two pathways, the oxidation to NO_3_^−^ and the reduction to NH_4_^+^, and the reduction way was more efficient to reactivate the catalyst. The two adsorption configurations of NO on Pt surface, the linearly adsorbed NO (NO_L_ in Figure 7g) and bridged‐bonded NO (NO_B_ in Figure 7g), exhibited different threshold potential during the oxidation/reduction processes.

In the cases that the contaminants proceed dissociative adsorption on the surface, in situ techniques could play an even more important role for the proposal of mitigation strategies. For instance, with the assistance of in situ XAS and electrochemical test methods, Swider‐Lyons’ group^[^ ^100^ ^]^ have revealed that the adsorption of chlorobenzene generated Cl^−^ on the catalyst surface which can induce permanent performance loss of a fuel cell. Consequently, they proposed that liquid water scavenging can remove the Cl^−^ efficiently and recover the cell performance.

## 4 Development of High‐Performance Catalyst Guided by In Situ Studies

The development of advanced ORR catalysts has attracted increasing attention in the past decades due to its importance to the commercialization of PEMFCs. Various reviews of the progress and perspectives in developing ORR catalysts have been published.^[^ ^101^, ^102^, ^103^, ^104^ ^]^ In this section, we focus on the development of catalysts with high ORR activity, anti‐oxidation activity, and SO_2_‐resistance, guided by the mechanisms demonstrated above and some other in situ studies.

### 4.1 Development of Highly Active Catalyst

#### 4.1.1 Principles for the Optimization‐ Ligand Effect and Strain Effect

**Figure 8:**
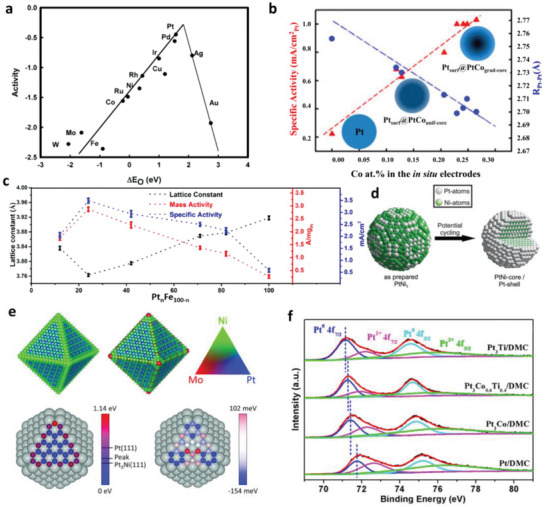
a) Trends in oxygen reduction activity plotted as a function of the oxygen binding energy. Reproduced with permission.^[^ ^66^ ^]^ Copyright 2004, American Chemical Society. b) The linear composition‐strain‐activity correlation for Pt_x_Co/C catalysts, also shown is the atom distribution in the catalyst; the darker color represents the higher Co concentration. Adapted with permission.^[^ ^107^ ^]^ Copyright 2015, American Chemical Society. c) Mass activity, specific activity, and lattice constant versus Pt composition in Pt_n_Fe_100‐n_ twisty nanowires (TNWs). Reproduced with permission.^[^ ^42^ ^]^ Copyright 2020, American Chemical Society. d) Schematic representation of Pt_3_Ni alloy NP evolution during electrocatalysis. Reproduced with permission.^[^ ^108^ ^]^ Copyright 2013, American Chemical Society. e) The average site occupancies of the second layer of the Ni_1175_Pt_3398_ nanocrystal (upper left) and the Mo_73_Ni_1143_Pt_3357_ nanocrystal (upper middle) at 170°C as determined by means of a Monte Carlo simulation. The calculated binding energies for a single oxygen atom on all fcc and hcp sites on the (111) facet of the Mo_6_Ni_41_Pt_178_ nanocrystal (lower left). The change in binding energies when a Ni_47_Pt_178_ nanocrystal is transformed to a Mo_6_Ni_41_Pt_178_ nanocrystal (lower right). Mo doping could decrease the oxygen binding energies at sites closer to Mo that bind oxygen too strongly while increase the oxygen binding energies at sites closer to the center of the (111) facet that bind oxygen too weakly. Reproduced with permission.^[^ ^109^ ^]^ Copyright 2015, the American Association for the Advancement of Science. f) Pt 4f XPS spectra of Pt_3_Ti/DMC, Pt_3_Co_0.6_Ti_0.4_/DMC, Pt_3_Co/DMC, and Pt/DMC. Reproduced with permission.^[^ ^110^ ^]^ Copyright 2022, American Chemical Society.

According to the aforementioned ORR mechanism, the reaction process involves several O‐containing intermediates, byproducts, and products. The adsorption energies of those O‐containing species, especially O and OH, play a decisive role in the activity of catalyst, which has been investigated by a classic DFT calculation.^[^ ^66^ ^]^ The calculation results revealed a volcano‐shaped relationship between the rate of ORR and the O adsorption energy, as shown in **Figure** **8**a. For the metals that bind oxygen too strongly (to the left of the maximum), the ORR rate was limited by the removal of adsorbed O and OH. For metals that bind oxygen too weakly, the rate was limited by the dissociation of O_2_ or the transfer of electrons and protons to adsorbed O_2_ (depending on whether ORR occurring through dissociative pathway or associative pathway). The result explained why Pt was the best ORR catalytic metal. However, in comparison with the theoretical optimal one, the binding energy between Pt and oxygen was still a little too strong. Another calculation result suggested that alloying Pt with other metals can be employed to improve the activity of Pt by adjusting the binding energies of oxygenated intermediates.^[^ ^105^ ^]^

Based on an in situ XAS measurement by Mcbreen's group,^[^ ^106^ ^]^ it is suggested that the change of the binding energies of oxygenated intermediates could be realized via two effects: the ligand effect or the electronic effect that refers to the bonding between Pt and the alloy metals, and the strain effect that refers to the changes in the Pt–Pt distance on the surface originating from the lattice mismatch. They have collected the in situ XANES spectra of PtCr/C, PtFe/C, PtCo/C, and PtNi/C alloy catalysts as well as Pt/C catalyst at Pt L_3_ edge. The d‐band vacancies of the Pt 5d‐orbital for the alloy catalysts were higher than that for Pt/C. Since the measurements were applied under the potentials where no adsorption of oxygenated species occurred, the variation of the d‐band vacancies was attributed to the ligand effect from the alloy metals. Besides, they have also observed the shorter Pt–Pt bond in the alloy catalysts with in situ EXAFS, suggesting the existence of a contraction strain effect. A combination of the ligand and strain effect reduced the Pt–O affinity of those alloy catalysts to the value closer to the theoretical optimal one, resulting in two‐ to threefold ORR activity enhancement as compared with Pt/C.

Monitoring the two effects with in situ methods could provide the real activity variation trends as well as feasible guidelines for the optimization of catalytic activity. The strain effect is relative to lattice mismatch that the lattice constant and Pt–Pt bond length can be regarded as the descriptors for the strain effect, which could be directly monitored with in situ EXAFS and XRD, respectively. For instance, Mukerjee's group^[^ ^107^ ^]^ have measured the Pt–Pt bond length (R_Pt‐Pt_) with in situ EXAFS and found that the ORR activity of the Pt_x_Co/C and Pt/C catalyst was linearly related to R_Pt‐Pt_. Meanwhile, R_Pt‐Pt_ decreased linearly with the increase of in situ Co/Pt atomic ratio obtained with in situ XAS. Thus, a linear composition‐strain‐activity correlation was established, as shown in Figure 8b, indicating that the strain effect is controllable by changing the atomic composition of the alloy catalysts. Zhong's group^[^ ^42^ ^]^ have synthesized a series of TNW Pt_n_Fe_100‐n_ with different n and investigated the relationship between the mass/specific activity and n as well as the lattice constant obtained with ex situ XRD, as depicted in Figure 8c. However, the composition of the catalysts always changed with the operation conditions. Thus, they further monitored the lattice constants and the composition of the catalysts with in situ HE‐XRD/PDF, providing the real activity variation trends during the operation of a PEMFC.

The ligand effect originating from the electronic interactions would induce modification of the electronic structure of Pt, and thus be monitored with in situ XANES.^[^ ^106^ ^]^ As compared to strain effect, ligand effect is much more difficult to be precisely monitored with in situ techniques because there are too many factors, including adsorbates, operating temperatures and potentials, that might impact the electronic structure of Pt during operations. To the best of our knowledge, there is no efficient technique that could isolate the ligand effect in the complicated circumstances. Methods such as XAS and XPS that could probe electronic structure in ultrahigh vacuum might be more appropriate to study ligand effect precisely in an ex situ way.

#### 4.1.2 Binary Pt‐Based Alloy Catalysts

As demonstrated above, the design and synthesis of PtM(M is usually a transition metal or lanthanide metal) binary alloy catalysts to promote the intrinsic ORR activity of Pt is a typical case of execution of the strain and ligand effect, which has made some achievements that PtCo/C catalyst has already been applied in commercial PEMFC vehicles.^[^ ^111^, ^112^ ^]^ In addition to the enhanced intrinsic activity of Pt, another advantage of PtM catalyst is the promoted Pt mass activity by reducing the consumption of Pt, especially in the M‐core/Pt‐shell structure.

The results of in situ tracking of the atomic distribution in some PtM catalysts during electrochemical processes indicate that the random alloyed PtM catalysts (in which the multiple metal components exhibit random atomic distribution) would perform a dealloying behavior due to the leaching of M when exposed in the acidic cathode. Strasser's group^[^ ^108^ ^]^ have tracked the atomic‐scale structure evolution of PtNi_x_/C during potential cycles with in situ surface X‐ray scattering (SXS). Disordered PtNi_3_ NPs showed a dealloying behavior during the electrochemical process with leaching Ni and evolving into PtNi‐core/Pt‐shell NP structure (as shown in Figure 8d). Chlorkendorff’ s group^[^ ^113^ ^]^ have observed a similar dealloying process in PtY alloy catalyst with in situ APXPS under the operation conditions in the cathode of a PEMFC. These in situ studies indicated that the M‐core/Pt‐shell structure exhibited higher stability than the random alloyed structure because the stable Pt shell could protect M in the core. Thus, various synthetic methods such as the galvanic displacement of an underpotentially deposited (UPD) foreign metal, thermal annealing, acid etching, etc.^[^ ^101^ ^]^ have been proposed to prepare PtM catalysts with core–shell structure. It has been confirmed with in situ techniques that the as‐prepared catalysts always maintain the core–shell structure during operations. For example, Adzic's group^[^ ^32^ ^]^ have synthesized core–shell PtPd/C catalyst with Cu‐UPD method and then the stability of the structure during electrochemical processes was confirmed with in situ EXAFS. Either synthesized on purpose or originating from the dissolution of surface M under the cathode condition, the core–shell structure could efficiently promote both the mass activity and intrinsic activity of Pt because only the most outer layers of the NPs are composed of Pt and the strain/ligand effect from M has been maintained well. Nevertheless, fine control of the Pt shell thickness is needed when synthesize the core–shell structure since too many Pt outer layers would eliminate the strain effect and shield the ligand effect from the M core.

In general, the in situ studies of PtM/C catalysts revealed that the ligand and strain effect could promote the intrinsic ORR activity of Pt, while the M‐core/Pt‐shell structure could further improve the Pt mass activity by reducing Pt usage. The in situ techniques provide visualized descriptors for the electronic and structural properties which could help deepen the understanding of the composition/structure‐activity relationship and further provide efficient assistance for the rational design of highly active ORR catalysts.

#### 4.1.3 Ternary Pt‐Based Alloy Catalysts

Although PtM binary alloy catalysts have exhibited higher activity than Pt/C catalyst, their performance is still unsatisfactory in the real applications. Further efforts have been exerted to develop ideal ORR catalysts on the basis of PtM catalyst, among which the introduction of an additional metal to form ternary alloys is a promising strategy. With in situ techniques, the role of the third metal has been intensively investigated, providing guidelines for the design of ternary Pt‐based catalyst.

Huang and Duan's group^[^ ^109^ ^]^ have made fruitful achievements in developing higher‐performance ternary Pt‐based alloy catalysts by doping a series of transition metals onto the surface of octahedra Pt_3_Ni/C. Among them, Mo‐Pt_3_Ni/C exhibited the highest specific and mass activity and DFT calculations revealed that the doped Mo could fine‐tune the chemical and electronic properties of the surface, shifting the oxygen binding energies at specific sites closer to the theoretical optimal value (as depicted in Figure 8e). A further in situ XAS and DFT calculation study^[^ ^114^ ^]^ revealed that Mo surface dopant could enrich and stabilize the subsurface Ni, thus the ligand and strain effect from Ni were intensified and preserved, inducing the enhancement of ORR activity. Besides, the electron transfer from Pt to Mo oxides also facilitated the ORR activity of Mo‐PtNi/C. Based on the in situ studies, the introduction of a third metal in PtM alloy catalyst could enhance the catalytic activity through two pathways: the direct electron transfer between the metal and Pt, and the modulation of M which further optimize the electronic structure of Pt, both of which have been reported separately. For instance, Liao's group^[^ ^110^ ^]^ have reported a ternary alloy catalyst, Pt_3_Co_0.6_Ti_0.4_/DMC (ZIF‐8‐derived mesopore carbon), in which the third metal Ti exhibited direct electron transfer to Pt, inducing the higher ORR activity of Pt_3_Co_0.6_Ti_0.4_/DMC than Pt_3_Co/DMC. The results of an ex situ XPS measurement showed that the Pt 4f binding energy shifted more negatively in Pt_3_Co_0.6_Ti_0.4_/DMC than in Pt_3_Co/DMC (as depicted in Figure 8f), indicating more electron transfer from Ti than Co to Pt. Adzic's group^[^ ^115^ ^]^ have synthesized a PtIrNi/C catalyst with a Ni‐core/Ir‐shell/Pt‐shell structure as measured with in situ XAS, ex situ TEM, and DFT calculations. It was confirmed that the introduction of Ni favored the anti‐segregation of Ir to the Pt‐shell and further modulated the oxygen binding energy of PtIrNi to a more suitable value for ORR.

However, although the authors have reported the direct and indirect pathways separately, the two pathways would work in parallel in most cases since the three kinds of metal atoms are not strictly separated in a ternary alloy catalyst. In general, the introduction of a third metal in PtM alloy catalyst could boost the catalytic activity but at the same time makes the mechanism study arduous so that research techniques including in situ characterizations and theoretical calculations are required to unravel the role of the third metal unambiguously.

### 4.2 Development of Highly Durable Catalysts

#### 4.2.1 Anti‐Oxidation Catalysts

According to the aforementioned Pt oxidation mechanism, *OH plays an important role in the oxidation of Pt. The development of anti‐oxidation electrocatalyst could be afforded by inhibiting the adsorption of OH through the blockage of the active sites with other species. The blockage species could be an alloy metal such as Mo in MoPtNi/C.^[^ ^114^ ^]^ The Mo atoms mainly adsorbed at the particle edge and vertex sites which were also the favored adsorption sites for OH. The Mo oxides (Mo^4+^/Mo^6+^) existed within the entire fuel cell operating potential range and thus inhibited the adsorption of OH and the consequent oxidation at these sites. An external modification species such as liquid ion might also help to prevent the generation of Pt–OH. Uchimoto's group^[^ ^116^ ^]^ have investigated the in situ XAS spectra of the ionic liquid‐modified Pd‐core Pt‐shell catalyst as well as that of the naked catalyst. It was revealed that the modified catalyst displayed smaller 5d orbital vacancies than the naked one, indicating a lower coverage of oxide species on the surface and subsequently an alleviation of the oxidation of Pt.

On the other hand, pushing the generation of Pt–OH to the potentials higher than the cell operating potential is another way to develop anti‐oxidation catalysts. Adzic's group^[^ ^117^ ^]^ have collected the in situ XANES spectra at the Pt L_3_ edge for both Pt/C and Au‐cluster‐modified Pt/C under varying potentials. The results reflected that the oxidation of Pt commenced at higher potentials for AuPt/C than that for Pt/C. Hyeon's group^[^ ^118^ ^]^ suggested that by alloying with Fe, Pt atoms became more oxidation‐resistive because of the higher oxidation potential than pure Pt. Moreover, encapsulating PtFe/C with N‐doped carbon shell by thermal annealing can stabilize Fe in the NPs and maintain the oxidation‐resistance of the catalyst, which was confirmed with in situ XANES, EDS, and first principle calculations. Although these catalysts did not exhibit higher oxidation potentials than the cell operating potential, they indicated the possibility that by alloying with another metal, the oxidation of Pt would proceed at higher potentials.

According to an in situ study from Liu's group,^[^ ^93^ ^]^ introducing an alloying element that can be oxidized instead of Pt, termed the sacrifice method, is also efficient to inhibit the oxidation and the following dissolution of Pt catalyst. They have compared the oxidation of PtNi/C and Pt/C in the cathode of a fuel cell with in situ XAS. The XANES spectra collected at Pt L_3_ edge at different potentials showed that the content of Pt in PtNi/C catalyst was higher than that of Pt/C at all potentials while the Ni element mainly exhibited an oxidized state and performed a sharp decreasing in content, which meant that the oxidation of Pt was significantly hindered by the oxidation of Ni.

Besides the oxidation of Pt, the oxidation of the carbon support (as shown in Equation 7) is also noteworthy which would further cause Pt NP detachment. To circumvent the issue, some alternative anti‐oxidation materials could be applied as the support. For example, Strasser's group^[^ ^119^ ^]^ suggested that ruthenium‐titanium mixed oxide (RTO) was an ideal anti‐oxidation support that showed high durability during ADTs according to the results of in situ HE‐XRD and online inductively coupled plasma‐mass spectroscopy (ICP‐MS).

#### 4.2.2 SO_2_‐Resistant Catalysts

Besides the regeneration protocols mentioned in Section 3.2.3, the rational design of poisoning‐resistant catalysts is also helpful to eliminate the poisoning effect. Two rules can be considered while design SO_2_‐resistant ORR catalysts: increasing the adsorption barrier of SO_2_ on Pt surface and accelerating the oxidation of SO_2,ad_ to remove it.

To develop SO_2_‐resistant catalyst with increased adsorption barrier of SO_2_, some Pt alloy catalysts, including PtRu^[^ ^120^ ^]^ and PtMo,^[^ ^121^ ^]^ have been developed. The introduction of alloying metal into Pt catalyst modifies the electronic structure of Pt, weakening the interaction between Pt and SO_2_, thus promotes the SO_2_‐resistance. The modification effect has been probed with in situ NAP‐HAXPES, as depicted by Yokoyama's group.^[^ ^122^ ^]^ They has confirmed that Pt_3_Co/C exhibited lower S adsorption after ADTs than Pt/C due to the more negative charge of the Pt surface in Pt_3_Co/C.

To accelerate the oxidation of SO_2,ad_ on Pt surface, one could search for threads in the SO_2_OR mechanisms as depicted in Section 3.2.3. With in situ FTIR, de Torresi's group^[^ ^123^ ^]^ highlighted the importance of S_2_O_6_^2−^ in Pt catalyzed SO_2_OR as the key intermediate and suggested that Pt(100) was the most active face which possessed an appropriate interaction strength with S_2_O_6_^2−^ in comparison with Pt(111) and Pt(110). The results indicates that a Pt‐based catalyst comprising more Pt(100) facet might be developed as SO_2_‐resistant catalyst. However, Pt(100) has been confirmed to exhibit poor ORR activity due to the strong *OH binding energy, making Pt(100)‐enriched catalyst not worth to be considered. As the oxidant species, *OH is another important intermediate during SO_2_OR. The enrichment of *OH during the reaction might facilitate the oxidation process. For instance, metal oxides with abundant hydroxyl groups on the surface, such as TiO_2_ and WO_x_, have been introduced to Pt catalyst and successfully accelerated the oxidation of SO_2_.^[^ ^124^ ^]^ On the other hand, modulating the generation potential of *OH to a more suitable place where SO_2_OR occurs would efficiently accelerate the oxidation. Swider‐Lyons and coworkers^[^ ^125^ ^]^ have provided clear understanding of the sulfur removal mechanism on Pt_3_Co/C electrode with in situ XANES measurement. The Co ligand in Pt_3_Co weakened the Pt–OH and Pt–S bonds, pushing OH adsorption to higher potential and the oxidation of SO_2_ to lower potentials. It allowed the OH adsorption and sulfur oxidation processes to occur at similar potentials which facilitated the latter because Pt–OH was the oxidant for it. On account of the modulation, Pt_3_Co/C can be more quickly and completely recovered after SO_2_‐poisoning through SO_2_OR as compared with Pt/C. Combined with the electronic modulation effect depicted in the previous paragraph, Pt_3_Co/C possesses both the higher SO_2_ adsorption barrier and the faster recovery rates. Furthermore, the weakened Pt–OH bonds indicate the higher ORR activity and anti‐oxidation activity than Pt/C. All the advantages above have made PtCo/C an ideal ORR catalyst in practical PEMFC applications, that might explain why it has been applied in industry.

## 5 Conclusions and Future Perspectives

As one of the bottleneck problems for the development of PEMFCs, the sluggish kinetics and complicated mechanisms of ORR at PEMFCs’ cathode have drawn extensive attention over the past several decades. In situ techniques as well as theoretical calculations have played pivotal roles in the study of the ORR mechanism by monitoring the intermediates and observing the evolution of catalysts. From a practical perspective, the failure mechanism of ORR catalysts, including the NP degradation of the catalyst under ADT conditions at the cathode, the oxidation of the catalyst under high potentials (induced by the automotive operations such as startup‐shutdown), and the poisoning of catalyst by the ambient contaminants, are of great importance and have been deeply studied with in situ techniques and calculations. Furthermore, the in situ studies of those mechanisms and some other in situ studies have provided crucial guidelines for the development of ORR catalysts with high activity, robust durability, and favorable toxic‐resistance that facilitate the commercial application of PEMFCs.

Despite the impressive progress achieved in light of in situ studies of ORR, there are still some challenges and opportunities for the development of the in situ techniques and theoretical calculations in the future:

1) The in situ cells for some characterization methods, such as IR and Raman, should be designed to be more realistic that can mimic the operating conditions of a real fuel cell. The in situ IR and Raman studies are now applied in the half‐cell which possesses enormous difference from a real fuel cell. The lack of a real in situ cell for IR and Raman might be assigned to the weak sensitivity of IR and Raman while the real fuel cell has complicated components including membrane and gas diffusion layer (GDL) that may produce a lot of interferences for the detection. Thus, the enhancement of IR and Raman sensitivity is also a direction that needs to work on for a more realistic in situ study.
2) The temporal resolution of the characterization techniques should be improved in future to capture the intermediates with short lifetime. For IR and Raman, it takes tens of seconds to gain a spectrum while for XRD/XAS, the time varies from minutes to hours. Even the most advanced QXAFS needs milliseconds for one spectrum. However, the reaction intermediates, especially those in the rate‐determining step, generally have a lifetime of picoseconds. The intermediates detected with in situ techniques at present are the stable ones in most cases, which cannot provide information of the whole reaction. Besides, the improvement of temporal resolution is also necessary to gain an in‐depth understanding of the reaction dynamics.
3) Multiscale in situ techniques for the full lifecycle study of a real fuel cell are needed to gain a full landscape of the cell evolutions during the operation which facilitates the optimization of the whole system of a fuel cell. Most of the in situ studies of ORR at present are aimed at the catalyst, the GDL, or the membrane independently and only provide a single piece of information. The development of in situ full lifecycle study techniques is of great practical significance for the real production and optimization of PEMFCs. Those techniques may include a combination of various in situ techniques, such as a couple of in situ XAS and in situ vibrational spectroscopy to gain the structure information as well as the intermediate information simultaneously. The multiscale characterization techniques should be able to monitor all the components of a fuel cell including the catalyst layer, the membrane, and the GDL simultaneously and reveal the interference as well as synergistic effect among them. This also needs the development of in situ techniques with high spatial and temporal resolution.
4) Although a large number of theoretical calculations have been used in combination with the in situ techniques to study the underlying mechanisms of ORR, there are still some development opportunities for theoretical methods. DFT calculation methods considering of explicit gas, catalyst, and solvent molecules in a fuel cell can capture the integral information of the interaction between the catalyst and the support, the catalyst and the solvent, as well as the interaction among the gas molecules, which facilitates the precise interpretation of the in situ experimental data. Those calculation methods may need large computation resources and are still underdeveloped. The blossoming in situ characterization techniques put forward higher requirement for calculation methods. For instance, efficient multi‐modal data analytic methods are crucial for the multiscale in situ techniques that generate massive data in a short period of time. Machine learning is another development direction for theoretical methods, which can efficiently help dealing with mass in situ experimental data.

## Conflict of Interest

The authors declare no conflict of interest.
